# Design and Evaluation of an Acoustic Modem for a Small Autonomous Unmanned Vehicle

**DOI:** 10.3390/s19132923

**Published:** 2019-07-01

**Authors:** Siyuan Zheng, Feng Tong, Bin Li, Qiuyang Tao, Aijun Song, Fumin Zhang

**Affiliations:** 1 The Key Laboratory of Underwater Acoustic Communication and Marine Information Technology of the Minister of Education, Xiamen University, Xiamen 361005, Fujian, China; 2 School of Electrical and Computer Engineering, Georgia Institute of Technology, Atlanta, GA 30332, USA; 3 Department of Electrical and Computer Engineering, The University of Alabama, Tuscaloosa, AL 35487, USA

**Keywords:** doppler, AUV, acoustic modem, direct sequence spread spectrum (DSSS)

## Abstract

Design of underwater acoustic (UWA) modems for compact-sized, underwater platforms such as autonomous underwater vehicles (AUVs) is challenging because of the practical requirement to keep an engineering balance between the performance and the system overhead. Considering this type of mobile communication scenario, Doppler spread as well as the multipath draws substantial attention in implementing the system’s design and engineering. Specifically, for a small AUV, the large computational complexity of real-time resampling for the classic Doppler correction poses significant difficulty for the limited capability of the low-cost processor. In this paper, by adopting an adjustable AD (analog-to-digital) sampling rate, a Doppler compensation approach is proposed to enable low-complexity hardware implementation. Based on this, a direct sequence spread spectrum (DSSS) acoustic modem is designed for a low-cost, small-sized AUV. Meanwhile, the performance evaluation of this acoustic modem is conducted in terms of the robustness upon varying Doppler as well as AUV integration. Finally, experimental results performed on a commercial, small-sized AUV under different speeds are reported to verify the effectiveness of the proposed acoustic modem.

## 1. Introduction

With the rapid development in underwater information acquisition and transmission, the small underwater autonomous mobile platform is drawing more and more attention from the fields of marine environmental monitoring, ocean sampling, and oceanic engineering because of its low-cost, convenience, and capability for dense deployment. Underwater acoustic communication is urgently required for many autonomous underwater vehicle (AUV) applications to allow transmission of data and control among each other [1]. However, it has been well recognized that multipath expansion, time-varied fading, and Doppler spreading in both time and frequency domains are challenging topics for underwater acoustic communication.

For the specific application in small AUVs, because of the relatively short distance as well as low data rate requirement, the problems of low signal noise ratio (SNR) or channel multipath can be mitigated by adopting a suitable modulation manner such as spread spectrum. Thus, in many cases the Doppler spread generated by AUV motion becomes the most severe issue [2] to be addressed by modem design.

A significant amount of research has been conducted on Doppler estimation and correction. Johnson et al. [3] proposed to calculate the cross-correlation between a branch of local Doppler reference signals and the received signal, the highest correlation among which corresponds to the real Doppler. To reduce the number of correlation calculations needed for a Doppler search, Sharif et al. [4] adopted two LFM (linear frequency modulation) signals spaced with a fixed period to produce two correlation peaks, the interval of which offers an indicator for the bulk Doppler. The proposed Doppler estimator proved to be robust despite the complex channel responses. Tu et al. [5] designed a multiple-resampling (MR) scheme, where each MR branch matched the Doppler factor of a particular propagation path.

Among other areas of progress, He et al. [6] proposed an accurate Doppler shift estimation method, which automatically matched underwater acoustic multipath channels. This Doppler shift estimation scheme also featured high channel adaptability. Yue et al. [7] proposed a Doppler estimation and compensation method for fast-moving transceivers by incorporating the Doppler invariance of hyperbolic frequency modulation. Cui et al. [8] presented a Doppler estimation method using a cyclic prefix to achieve higher accuracy.

Compared with extensive and active research in different Doppler estimation strategies, there is a lack of investigation on the theoretical principle of Doppler correction, as it is straightforward that Doppler compensation can be effectively achieved by resampling after estimating Doppler with various approaches. While it is easy and convenient to conduct offline resampling in software, from the viewpoint of hardware implementation, real-time resampling still poses significant difficulties to the engineering application of an acoustic modem, mainly because of the computational complexity involved. For small AUVs where the power supply and processor calculation capability are strictly limited, reconstructing and resampling the frame signal, thus, poses challenges to the onboard electronics.

In this paper, a hardware Doppler mitigation approach is proposed in the modem design. By directly tuning the analog-to-digital (AD) sampling rate according to the Doppler estimate, our approach significantly reduces the computational load for its implementation on small AUVs. The proposed modem also utilizes a spread spectrum modulation scheme to enable robust, low data rate communication. Each signal frame begins with a synchronization signal that also serves as the Doppler probe. Once the synchronization is in lock, Doppler can be estimated to calculate the corresponding sampling rate to correct the Doppler spread. Then, the AD sampling rate is adjusted accordingly to directly compensate for the Doppler effects. Our approach enables the adoption of a low-cost and relatively simple processor for modem design; thus, it is well suited to small AUVs. A sea trial as well as a lake trial has been conducted to verify the effectiveness of the proposed acoustic modem.

## 2. System Design

### 2.1. Direct Sequence Spread Spectrum (DSSS) Modulation

**Figure 1:**
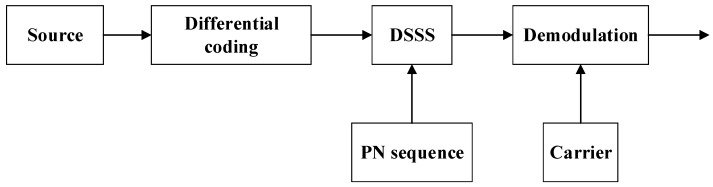
Block chart of the direct sequence spread spectrum (DSSS) demodulation. PN = Pseudo Noise.

As the principle of spread spectrum (SS) technology is well known, they are briefly reviewed here from an engineering viewpoint of the modem design. By directly modulating the information to be transmitted into a broad frequency band with a pseudorandom sequence and then recovering the transmitted information sequence through demodulation and de-spreading processes, a direct sequence spread spectrum (DSSS) is adopted to yield a strong anti-interference ability, a strong anti-multipath capability, and multiple user access capabilities and robustness under low SNR [9]. Moreover, differential coherent demodulation is employed to mitigate the time variations induced by UWA channels. The block diagram of DS-DBPSK (Direct Sequence-Differential Binary Phase Keying) modulation is shown in Figure 1.

**Figure 2:**
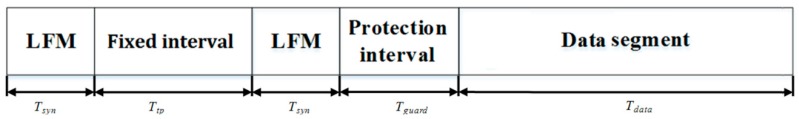
Frame structure of the modem signal. LFM = linear frequency modulation.

**Figure 3:**
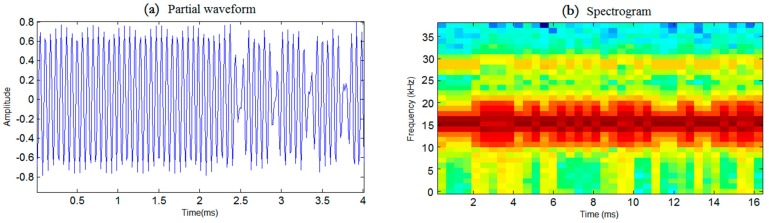
Partial waveform and spectrogram of a data symbol. (**a**) Partial wavform, (**b**) Spectrogram.

The frame structure of the system is displayed in Figure 2, which consists of two LFM signals and the data packet. While the first LFM signal acts as the synchronization header as well as the first reference for Doppler estimation, the second one is used as the second reference signal for Doppler estimation with a fixed interval between them. Considering the precision of Doppler estimation as well as the communication efficiency, the length of the fixed interval *T_tp_* is set to be ten times the length of the LFM signal *T_syn_*. Meanwhile, a protection interval was inserted between the second LFM signal and the following data packet to avoid interference, the length of which *T_guard_* equals the length of the LFM signal. The specific duration of each component of the frame structure is provided in Table 1. Shown in Figure 3a,b is the partial waveform as well as the spectrogram of a data symbol, respectively.

### 2.2. Doppler Estimation and Correction

As the estimation of Doppler is not the novel contribution of this work, a conventional and simple time- domain estimation approach is directly utilized to facilitate practical implementation. With two chirp signals located with a fixed interval $T_{tp}$ in the signal frame, as shown in Figure 2, the Doppler can be obtained by performing cross-correlation and then measuring the real interval $T_{rp}$ of two correlation peaks associated with these two LFM signals. As the Doppler time domain expansion is equivalent to the degree of signal contraction or expansion [10], the Doppler factor can be calculated according to the following equation:

(1) $$\Delta \approx \frac{T_{rp}}{T_{tp}}-1.$$

Generally, after estimating the Doppler, a resampling calculation can be directly used to compensate for it. As mentioned above, while it is pretty convenient to perform resampling in an offline manner by means of computation software such as Matlab, from the viewpoint of practical modem design the implementation of resampling poses significant difficulties for the microprocessor because of the real-time computational complexity involved. Considering that adjusting the AD sampling rate is equivalent to resampling the received signal, a novel Doppler correction approach, by directly tuning the AD sampling rate, is proposed to reduce the real-time computational complexity of the microprocessor and thus facilitate hardware implementation.

Specifically, the total duration of a frame is 6.8 s for the proposed modem, corresponding to 510,000 samples under a signal sampling rate of *f_s_* = 75 K sps. With the traditional principle of software resampling, the original signal sequence needs to first be up-sampled by an integer factor of *I*, and then the output sequence is calculated by a linear interpolation algorithm according to the following equation [11]:

(2) $$y(m+1)=(1-It_{m+1})y_{1}(m+1)+It_{m+1}Iy_{2}(m+1),$$

where y(m+1) is the output after resampling, and $y_{1}$ and $y_{2}$ are the adjacent points after up-sampling. One may conclude from the algorithm equations that the computational complexity of the resampling process algorithm is *O*($m^{2}$), which means a large calculation requirement considering the frame duration of the proposed system. Instead, by the proposed Doppler correction method of adjusting the AD sampling rate, the Doppler compensation can be directly fulfilled at the same time as the analog-to-digital conversion process; thus, no additional calculation is needed in the microprocessor for the Doppler correction. In other words, the proposed hardware resampling scheme will enable the adoption of a low-cost microprocessor with a relatively low calculation capability and small memory space.

### 2.3. Hardware Implementation of Analog-To-Digital (AD) Sampling Rate Adjustment

**Figure 4:**
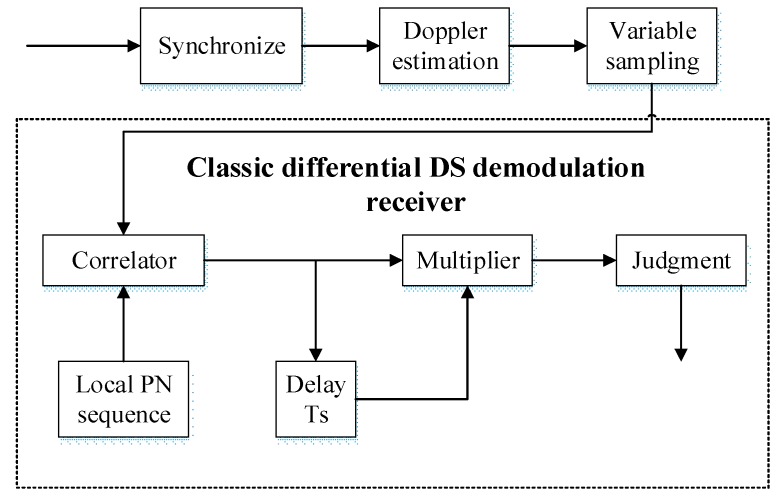
Illustration of the demodulation processing of the receiver.

The acoustic modem designed in this paper is based on the STM32F4 series processor of the Coetex-M4 core, which has the advantages of short cycle times and low power consumption under a clock speed of up to 168 MHz. As the Doppler is directly compensated by hardware resampling, the proposed system is capable of greatly reducing the complexity of the real-time calculation in the processor, thus facilitating hardware implementation and engineering integration in a small AUV platform [12,13]. To further improve the communication performance, convolutional coding is also adopted with a convolutional coding rate of 1/2. The detailed implementation of DSSS demodulation at the receiver is shown in Figure 4.

For the hardware Doppler correction method by tuning the AD sampling rate, the resolution of the AD sampling rate adjustment will determine compensation performance. In the proposed system, the internal ADC (Analog-to-Digital Converter) of the STM32F4 processor is directly used under the timer interruption mode; thus, the instant sampling rate *f_as_* is calculated by dividing the clock frequency *f_c_* with a timer parameter. With an *f_c_* = 84 MHz, the timer parameter is set to generate the instant sampling rate *f_as_* corresponding to the calculated Doppler according to the following equation:

(3) $$f_{as}=\frac{f_{c}}{1+\Delta }.$$

Specifically, implementation of the adjustable AD sampling rate is achieved by software to set the timer parameter of the processor according to the calculated Doppler. As there is a protection interval between the second LFM signal and the data segment, the calculation of Doppler as well as the adjustment of the AD sampling rate can be conducted during this period to ensure real-time implementation.

### 2.4. Software Design

**Figure 5:**
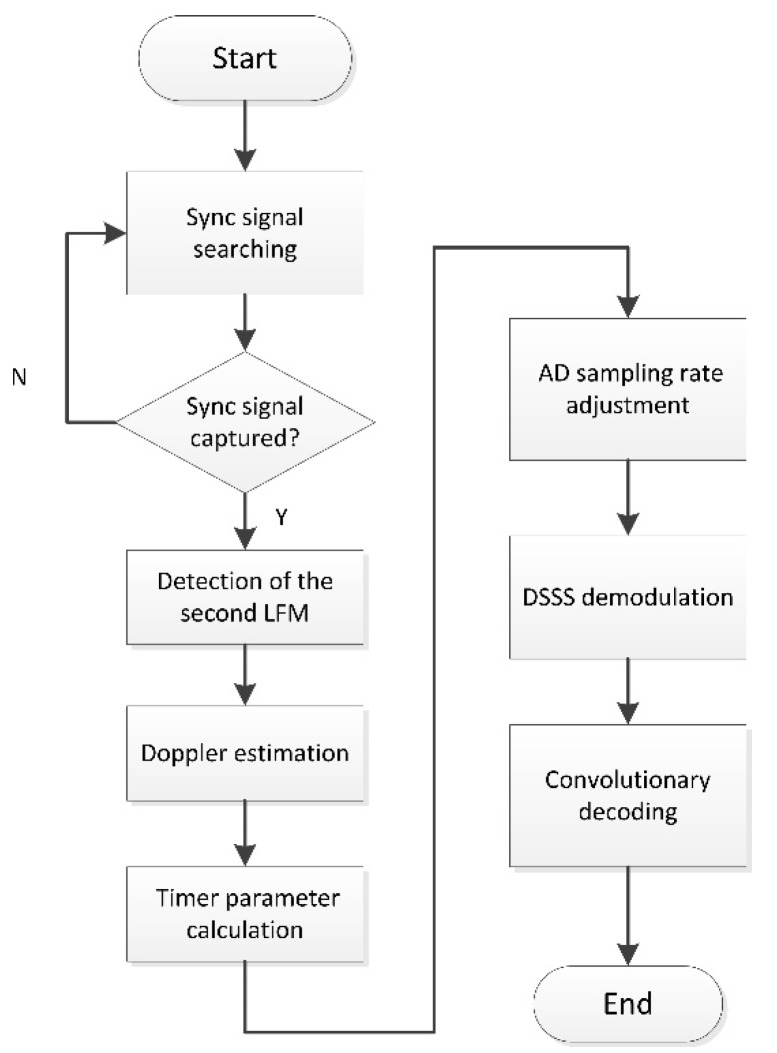
Software flow chart of the receiving.

As the software design of the transmitter is relatively straightforward for the community, we briefly introduce the software design of the AD sampling rate adjustable receiver to further illustrate the main contribution of this paper. The software flowchart of the receiver is presented in Figure 5, from which one can see the specific implemental procedures of the AD sampling rate adjustment.

At the beginning of the flowchart, successful detection of the first LFM signal indicates the capturing of synchronization and then triggers the detection of the second LFM signal. Afterward, the real interval $T_{rp}$ of two correlation peaks associated with these two LFM signals can be measured to facilitate Doppler estimation and timer parameter calculation. Then, the flowchart enters the timer interruption to trigger AD sampling, with the sampling rate determined by the timer parameter. With the adjusted AD sampling rate, the Doppler effects induced by the underwater acoustic channel can be compensated to guarantee the following demodulation procedures.

To ensure real-time implementation of synchronization capturing and Doppler estimation, a dual-buffer manner is adopted by dividing the memory space into a ‘ping’ buffer and a ‘pong’ buffer. With one buffer receiving data via ADC, the other buffer performs the cross-correlation calculation to detect and measure the arrival times of two LFM signals. The role of the two buffers will switch between ‘ping’ and ‘pong’ once the receiving buffer is full to guarantee real-time implementation.

Moreover, as the internal correlation function provided in the STM32 Digital Signal Processing function library will automatically save the end part of the previous correlation result to assemble with the current correlation result, continuity of the cross-correlation result can be ensured, even when the LFM signal is more or less equally spread over both buffers.

### 2.5. Hardware Design

**Figure 6:**
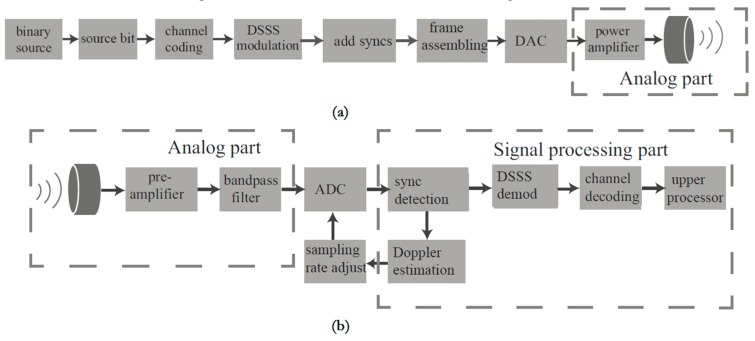
Block chart of the transmitter (**a**) and receiver (**b**). DAC = Digital-to-Analog Converter, ADC = Analog-to-Digital Converter.

The flow chart of the transmitter and receiver is provided in Figure 6. While the signal generation before DAC (Digital-to-Analog Converter) in the transmitter as well as the demodulation after ADC in the receiver is implemented in the processor in forms of digital signal processing, the purpose of the analog part in the transmitter and receiver is to guarantee efficient emission and high-quality signal reception, respectively.

Because the bandwidth of the proposed MODEM (Modulator and Demodulator) is within the audio frequency range, the general audio interface integrated circuit chip WM8978 is directly adopted for the DAC of the emission signal, which offers convenient control registers for the processor. The piezoelectric transducer is adopted in our design, the bandwidth of which is 13–18 kHz with omni-directivity in the horizon to facilitate the communication application. As most of the piezoelectric transducer exhibits capacitive characteristics, an inductance circuit is used as an electrical impedance matching network to improve the transmitting efficiency.

In the receiver, the low-noise AD603 integrated circuit chip is selected as the preamplifier because the gain of which can be controlled by extra voltage to provide accurate, pin-selectable gains of −11 to +31 dB with a bandwidth of 90 MHz, or +9 to +51 dB with a bandwidth of 9 MHz. For the band-pass filter, the continuous-time active filter MAX274 integrated circuit chip is employed as it provides four second-order sections to enable the implementation of an eighth-order band-pass filter.

## 3. Preliminary Results and Analysis

### 3.1. Experiment Setup

**Figure 7:**
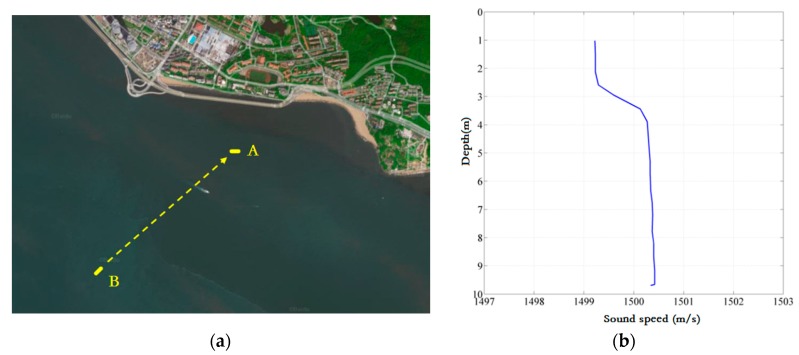
Experiment configuration and sound velocity profile of the 1st trial. (**a**) Configuration of the first trial. (**b**) Sound velocity profile of the first trial.

The experimental field of the first trial was the sea near Xiamen harbor, Xiamen, Fujian province, the configuration of which is shown in Figure 7. As displayed in Figure 7a, vessel A was anchored, while vessel B approached vessel A at a speed of about 3 knots. The distance between the two vessels was 1.2 km with a water depth of 10 m. The sound velocity gradient is shown in Figure 7b. The other configuration parameters of the modem are provided in Table 1.

### 3.2. Experimental Results and Analysis

**Figure 8:**
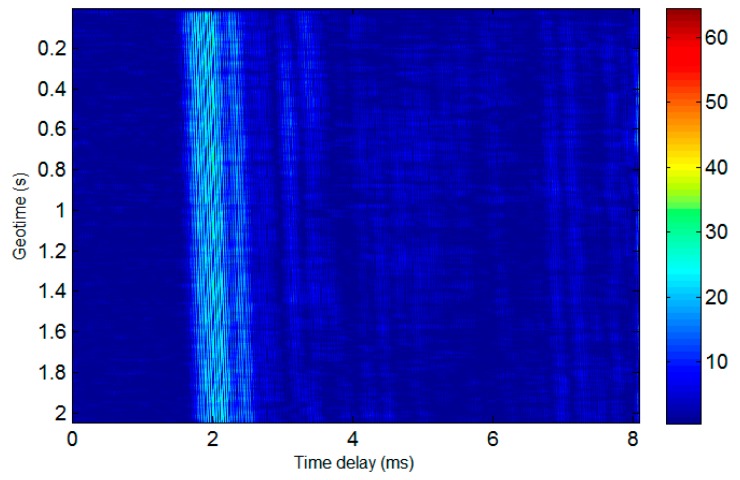
Response of the experimental channel for the first trial.

The time-varying response of the sea trial channel in the first experiment is shown in Figure 8. It can be seen that the channel had obvious multipath as well as Doppler shifts caused by the relative motion of the transceiver platform.

**Figure 9:**
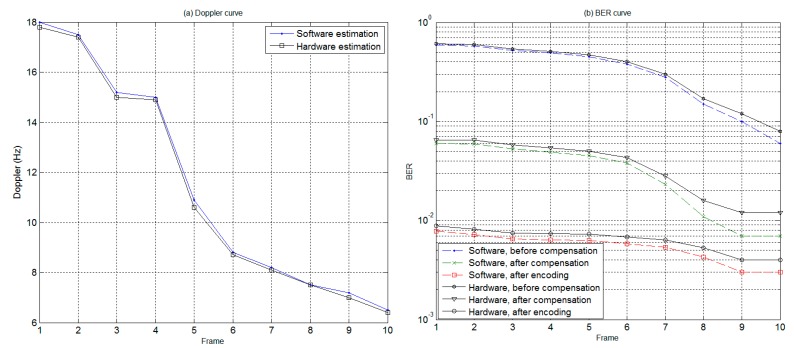
Doppler and bit error rate (BER) curves obtained with software and hardware approaches. (**a**) Doppler curve (**b**) BER curve.

In order to evaluate system performance, a total of ten frames of communication signals were collected for analysis between the software and hardware demodulation. For the software receiver, Matlab software was adopted for offline demodulation, while the proposed STM32 microprocessor hardware was used for real-time hardware demodulation with Doppler compensation implemented by tuning the resampling rate. The values of Doppler estimated by software and hardware are shown in Figure 9a, respectively, which reveals that the estimated results of both approaches were almost the same.

Shown in Figure 9b is the bit error rate curve corresponding to software and hardware receivers, wherein the solid line is a hardware demodulation error rate curve, and the dashed line is a software demodulation bit error rate curve. From the bit error rate (BER) results, it is evident that, with a higher calculation complexity, the performance of software demodulation is slightly better than that of hardware demodulation. The reason is that the precision of the hardware sampling chip, system clock, etc., is not as good as that of the software implementation.

In addition, Figure 9b also indicates that the BER after Doppler compensation is less than 0.06, and the communication performance is obviously better than that before compensation. Moreover, the adoption of channel coding further improves the BER to the level of under 0.01. In conclusion, by incorporating DSSS modulation and channel coding, the proposed Doppler compensation method based on an AD variable sampling rate is capable of achieving a good communication performance for a moving vehicle.

## 4. Autonomous Underwater Vehicle (AUV) Integration Results and Analysis

### 4.1. Experiment Setup

**Figure 10:**
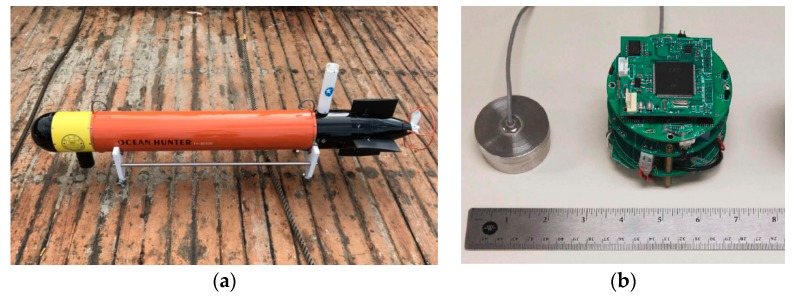
The autonomous underwater vehicle (AUV) and the acoustic modem module (**a**) The TH-050B AUV (**b**) The acoustic modem module.

**Figure 11:**
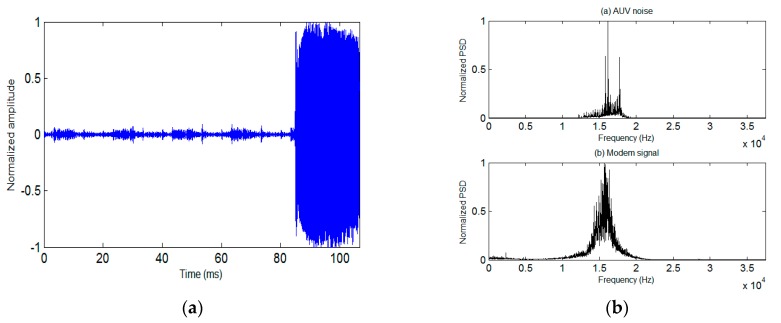

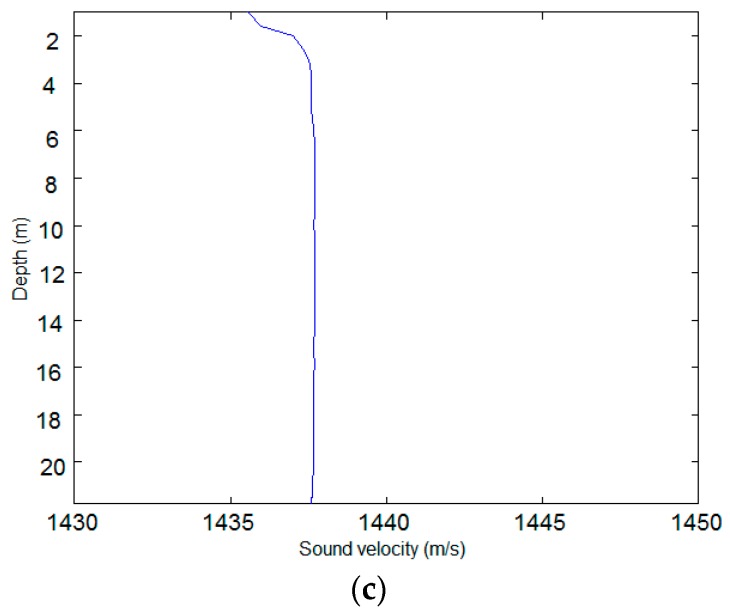
The waveform and associated spectrum of the communication signal. (**a**) Noisy waveform of modem signals. (**b**) Normalized power spectral density (PSD) of noise and signals. (**c**) Sound velocity profile of the second trial.

The trial field of the second experiment was Xingan River, Zhejiang province, China. To evaluate the performance of the proposed design in terms of engineering AUV integration, the acoustic modem was integrated onboard a small-sized TH-050B AUV. With a length of 1500 mm and a diameter of 180 mm, the adopted AUV is a typical low-cost commercial AUV with the purpose of being used for extensive marine missions. With the help of it, the effectiveness of the proposed modem onboard AUV under different velocities was evaluated. Shown in Figure 10a is the picture of the AUV, with the picture of the acoustic modem module displayed in Figure 10b. The sound velocity profile of the trial area is displayed in Figure 11c.

With the receiver at an anchored vessel, the AUV was controlled to transmit while moving at different speeds ranging from 1 to 3 knots under a depth of 3 m. The distance between the AUV and the vessel was about 200 m with a water depth of 25 m. The signal parameters of the modem were the same as those in the first trial.

### 4.2. Experimental Results and Analysis

**Figure 12:**
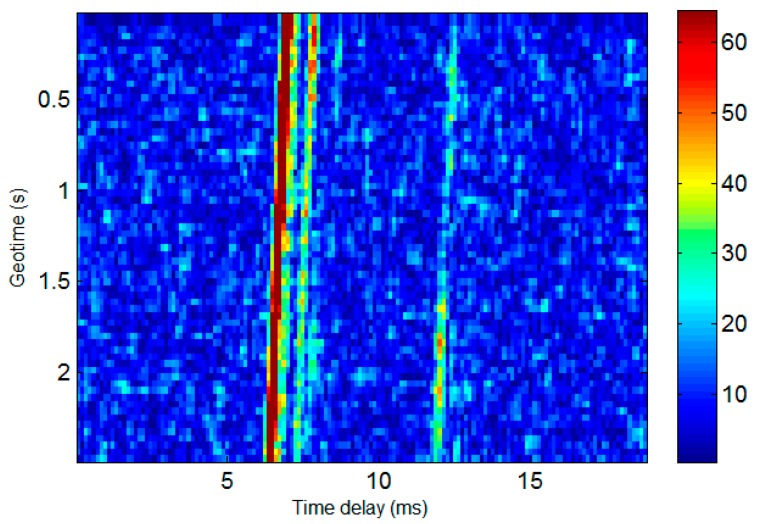
Response of the experimental channel for the second trial.

In Figure 11 the waveform and associated spectrum of the AUV communication signal collected during the second trial are presented, from which one can see the background noise of the moving AUV produced by the electromagnetic interference (EMI) of the motor. It is noticeable that the spectrum of AUV noise overlaps with that of the acoustic communication signal, leading to certain interference on communication performance. The channel response of the second trial is shown in Figure 12, which indicates the simultaneous presence of an apparent multipath and Doppler [14].

**Figure 13:**
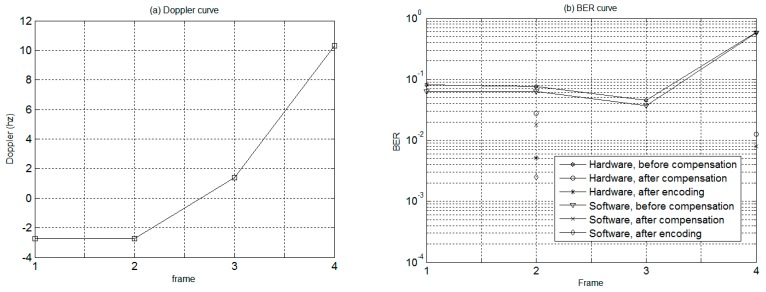
Doppler and bit error rate curves obtained by the modem onboard AUV. (**a**) Doppler curve, (**b**) BER curve.

The Doppler estimated by the modem is provided in Figure 13a, with the corresponding BER before and after Doppler compensation shown in Figure 13b. From Figure 13a we can observe that the Doppler generated by the AUV movement was in the range of −3 to 10 Hz. As displayed in Figure 13b, the proposed modem was capable of accommodating the small Doppler by generating a BER on the order of 10^−2^ without compensation. Meanwhile, the Doppler compensation can reduce the BER to zero under the Doppler of about 2 Hz, indicating error-free communication under a relatively small AUV speed.

Furthermore, under the Doppler of 10 Hz, while the BER approached 40% without Doppler correction, the BER performance of the proposed modem improved by 7% after Doppler correction and was further promoted to 3% after Doppler correction and channel encoding. Thus, the experimental results obtained by the modem verify the effectiveness of the proposed modem in Doppler mitigation when the AUV experiences a relatively fast movement.

## 5. Conclusions

This paper presents the design and performance evaluation of a compact modem for a small AUV, which adopts a hardware Doppler compensation method by directly tuning the AD sampling rate to greatly reduce implementation complexity. Meanwhile, a low data rate DSSS modulation scheme is adopted to further improve the performance under noise generated by the AUV itself. Experimental results of a mobile communication sea trial as well as a trial onboard the moving AUV verified the effectiveness of the proposed scheme, which has the potential of being used for small-sized and low-cost underwater vehicles.

## Figures and Tables

**Table 1:** Parameters of the proposed modem.

Spread Gain	63	Bandwidth	13–18 kHz
Carrier frequency	16 kHz	Bit per frame	384
Transducer directivity	Omnidirectional	PN code	M sequence
Bit rate	60	Modulation	DSSS
Diameter	80 mm	Height	100 mm
Channel encoding	Convolutional Code, Coding rate (1/2)	Default Sampling rate *f_s_*	75 ksps
Processor clock frequency *f_c_*	84 MHz	$T_{tp}$	226 ms
LFM signal Length $T_{syn}$	22.6 ms	$T_{guard}$	22.6 ms
Symbol duration	16.67 ms	$T_{data}$	6.5 s
